# Evaluation of the Heart Failure in Internet Patient Information: Descriptive Survey Study

**DOI:** 10.3390/ijerph18031047

**Published:** 2021-01-25

**Authors:** Kyoung Suk Lee, Yoo Mi Cho, Sung Hee Oh, Mi Sook Jung, Ju Young Yoon

**Affiliations:** 1 College of Nursing, Seoul National University, Seoul 03080, Korea; kyounglee@snu.ac.kr (K.S.L.); popymoreni@snu.ac.kr (Y.M.C.); 2 Research Institute of Nursing Science, Seoul National University, Seoul 03080, Korea; 3 Center for Human-Caring Nurse Leaders for the Future by Brain Korea 21 (BK 21) four Project, College of Nursing, Seoul National University, Seoul 03080, Korea; 4 College of Nursing, Chungnam National University, Daejeon 35051, Korea; cutestsh@naver.com (S.H.O.); msj713@gmail.com (M.S.J.)

**Keywords:** heart failure, eHealth, learning need

## Abstract

Patients with heart failure (HF) may not receive enough HF education from their clinicians throughout the course of the illness. Given that information is readily accessible on the Internet, patients with HF may seek HF information online. However, the relevance of online information for patients, the health literacy demand, and quality of the information is unknown. The purpose of this study was to compare the HF topics available online with topics HF patients perceived to be important and to evaluate the health literacy demand and quality of online HF information. The most popular search engines and a website that ranks the popularity of the websites were searched to identify websites with HF information. The health literacy demand and quality of the information were evaluated using the Patient Education Material Evaluation Tool for Print Materials and the DISCERN tool, respectively. First, the HF Patients’ Learning Needs Inventory (HFPLNI) was used to determine whether the websites included the 46 topics identified in this inventory. Patients with HF (*n* = 126) then completed the HFPLNI to rate the perceived importance on each topic. A chi-square test was used to compare the differences between the topics on the websites and those patients perceived to be important. Of the 46 topics, 39 were less likely to be included on the websites even though patients perceived that they were important topics. Information on the websites (*n* = 99) was not written could not be easily understood by patients and did not meet the overall health literacy demands of 58.0% and 19.8% of the patients, respectively. Only one-fifth of the websites were rated as fair to good quality. Online HF information had high health literacy demand and was poor quality with mostly generic HF information, which did not meet patients’ information needs. Websites need to be developed reflecting patients’ learning needs with low health literacy demand and good quality.

## 1. Introduction

With information readily on the Internet, it has become common practice for individuals to seek health information online [1,2]. People may seek online information to learn more about health conditions for themselves or others and/or to clarify information given by their healthcare providers [1,3]. Mobile health applications have also become widely available to support self-care with features that track activities and provide relevant information [4]. This information can influence patients’ decisions about their health behaviors [5,6].

Given that heart failure is a chronic condition, patients with heart failure should adhere to recommended self-care regimens, which entail a number of life-long, complex activities [7,8]. To successfully engage in self-care, patients need a comprehensive understanding of heart failure, its symptoms, and self-care [9]. However, patients may not receive comprehensive heart failure information throughout the course of the illness because heart failure education is not a routine practice in many heath care systems, such as in South Korea. Therefore, it is not surprising that patients’ need for information about their condition and management strategies is not met by their healthcare providers.

Another challenge for patients is the relevance of the information they find online. Although a vast amount of health information related to heart failure is available, much of it may be different from what the patients are searching for, even after they carefully choose relevant search terms. Even if patients find relevant websites addressing the topics they are interested in, the quality of the information might be inadequate or written at a health literacy level that exceeds the patients’ level. Cajita and colleagues (2017) found that the heart failure information found online written in English had only fair quality, and required readers to have a relatively high level of health literacy [10]. Orlowski and colleagues (2013) observed similar results about heart failure online information on 15 websites including limited credibility and readability [11].

Clinicians are responsible for educating their patients by providing relevant information and suggesting additional helpful websites [12]. However, it is important to evaluate the relevance of the topics, quality, and literacy levels of online heart failure information so clinicians can recommend the best information. To the best of our knowledge, only one study, Cajita and colleagues (2017) has examined online heart failure information, but they did not evaluate the online information topics [10]. In addition, because an enormous number of new websites are created every day, their study results are unlikely to reflect current online heart failure information. Therefore, the purpose of this study is to systematically review online heart failure information. The specific aims are to (1) compare heart failure online information topics with topics patients with heart failure perceived to be important, (2) evaluate the required health literacy demand and the quality of online heart failure information, and (3) compare the health literacy demand and quality of online heart failure information among the websites.

## 2. Methods

### 2.1. Study Design

This was a descriptive survey study to systematically review online health failure information.

### 2.2. Webpage Search

To identify websites containing heart failure information, the following terms were searched: heart failure, congestive heart failure, acute heart failure, chronic heart failure, cardiomyopathy, and cardiac dysfunction. These search terms were entered into the three most popular search engines used in Korea (Google, Naver, and Daum). Potential websites for this study were restricted to the first ten pages of results for each search term. We also used a website (www.rankey.com) that ranks websites based on Internet users’ traffic over 12 weeks in diverse categories. To identify websites including heart failure information, we hand-searched websites identified from Rankey.com in the following six categories: (1) medical and health, (2) clinics, (3) academic or tertiary medical centers, (4) drugs, (5) western and oriental medicine, and (6) health management.

#### 2.2.1. Inclusion and Exclusion Criteria

Since the focus of this study was online heart failure information targeting patients with heart failure, the following inclusion and exclusion criteria were selected. Inclusion criteria were that the website (1) focuses on heart failure; (2) provides information about any aspect of heart failure; (3) is freely available without a log-in; and (4) is written in Korean. Exclusion criteria included: (1) unable to view due to technical problems after three attempts; (2) includes advertisements and other promotional materials; (3) targets health professionals or clinicians; (4) information about heart failure of animals; or (5) materials written by individuals without a clinical background (e.g., chatroom discussions).

#### 2.2.2. Evaluation and Inter-Rater Reliability

A pair of trained research assistants rated the content on each website using validated tools to assess the heart failure information. The heart failure topics were rated based on the Heart Failure Patients’ Learning Needs Inventory (HFPLNI). Health literacy demand was rated using the Patient Education Material Evaluation Tool for Print Materials (PEMAT-P). The quality of information was rated using the DISCERN tool. The supervising researcher along with the research assistants thoroughly read the user manuals of the PEMAT-P and DISCERN, and protocol for categorizing topics using the HFPLNI. Any unclear items were discussed until consensus was reached. To ensure rating consistency, the research assistants first rated the same five websites independently. Discrepant item scores were discussed and resolved with the supervising researcher. If their rating consistency did not reach a minimum of 95%, an additional five websites were rated until the target percentage for consistency was reached.

### 2.3. Patient Recruitment

#### 2.3.1. Setting and Sample

Patients with heart failure were recruited from outpatient cardiology clinics affiliated with academic medical centers in Korea. Patients were eligible if they were 21 years or older, diagnosed with heart failure, and living independently. Patients were excluded if their condition was severe enough to be listed on the cardiac transplant list or had psychological or neurological conditions that could interfere with cognitive function (e.g., stroke).

#### 2.3.2. Procedure

After obtaining Institutional Review Board approval from all sites, physicians referred eligible patients to the researchers. Trained research assistants explained the purpose and procedure to eligible patients, and obtained written, signed, informed consent from each patient who agreed to participate in the study. After consent, the participants were asked to complete the HFPLNI to examine patients’ information needs related to heart failure. In addition, patients provided demographic information.

### 2.4. Measure

#### 2.4.1. Heart Failure Information Topics

The HFPLNI was used to measure patients’ perceptions of the importance of 48 topics related to heart failure information [13]. Of the 48 topics, two topics were specific to hospitalized patients, so these two topics were removed. Each topic was rated on a five-point scale ranging from 1 (not important at all) to 5 (very important). The patients who were recruited from outpatient clinics were asked to complete the HFPLNI to measure their perceived importance of the 46 heart failure information topics. The Cronbach’s alpha coefficient of the Korean version was 0.97 in a previous study [14]. The trained research assistants also used this instrument to assess whether or not the websites included the topics listed in the HFPLNI (yes/no).

#### 2.4.2. Health Literacy Demand of the Information

PEMAT-P was used to evaluate the health literacy demand of the heart failure information on the websites [15]. PEMAT-P has two subdomains: (1) the level of understandability measuring how well the written material is understood by health consumers from diverse backgrounds with varying levels of health literacy, and (2) the level of actionability, which measures how well a health consumer is able to identify what they need to do based on the information presented. This instrument includes 24 items (17 items for understandability and seven items for actionability) with a binary scale (agree or disagree). Of the 24 items, 10 items were rated as not applicable (e.g., no numbers or visual aids were included in the material). The total scores were computed by averaging the items that were rated as “agree”, and then multiplying the result by 100. A score greater than 70% indicated that the material was understandable and actionable. The validity and reliability of the Korean version of the PEMAT-P were supported [16].

#### 2.4.3. Quality of the Information

The quality of the heart failure information was evaluated using DISCERN [17]. The DISCERN tool consists of 15 items to judge the reliability and quality of the information, as well as the overall quality. Each item was rated using a five-point scale: a score of 5 indicates that the item completely fulfills the quality criterion, scores of 4–2 indicate that the item partially fulfills it, and a score of 1 indicates that the item does not fulfill it at all. The overall quality rating ranges from 1–5, with 2 or below indicating poor quality with serious shortcomings, 3 indicating fair quality, and 4 or above indicating good quality. Based on the total DISCERN score from the 15 questions, the websites were grouped into the following categories related to the content: excellent (63–75), good (51–62), fair (39–50), poor (27–38), and very poor (15–26). The psychometric property of the Korean version was previously established [18].

### 2.5. Statistical Analysis

Data were analyzed using SPSS version 25 (Armonk, NY, USA: IBM Corp). Descriptive statistics including frequency distributions, means, and standard deviations were used to describe patient characteristics in addition to HFPLNI, PEMAT-P, and DISCERN scores. Patient ratings on each topic in HFPLNI were recoded to a binary variable to test whether important heart failure education topics identified by patients were significantly different from heart failure information topics found on the reviewed websites in this study. If patients’ ratings on the degree of importance were 4 (important) or 5 (very important) points, they were coded as “important”; if patients’ ratings were 1 (not important), 2 (somewhat important), or 3 (moderately important) points, they were coded as “unimportant.” Analysis of variance with Scheffe correction was used to compare the health literacy demand and quality of information among the types of website publishers.

## 3. Results

### 3.1. Search Results and Characterics of the Websites

**Figure 1:**
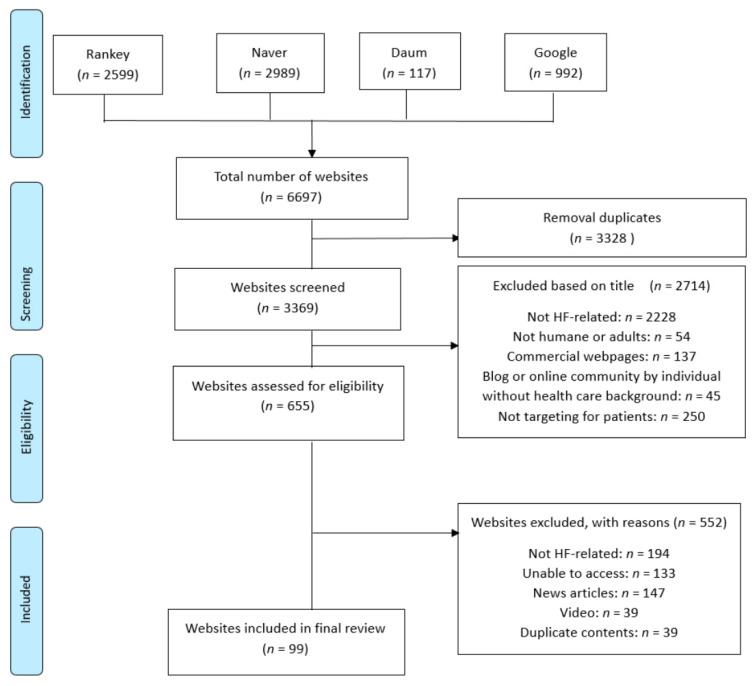
Search results using PRISMA flow diagram.

A total of 99 unique websites were included in this study (Figure 1). Among the 99 websites, seven were classified as government or professional organizations (e.g., Korean Heart Failure Society), 70 were affiliated with a hospital or clinic, 15 were commercial companies (e.g., pharmaceutical companies), and seven were physicians.

### 3.2. Characteristics of Patients

The average age of patients (*n* = 121) was 59 years (SD 12.99) with a range from 25–85 years (Table 1). The majority of the patients were male, had a high school education and above, and were categorized as New York Heart Association functional class I/II, indicating no or minimal functional limitations due to symptoms of heart failure. All patients took at least one medication related to heart failure.

### 3.3. Heart Failure Information Topics

#### 3.3.1. Topics Perceived to be Important by Patients

The most frequently reported topics that the patients rated as important or very important were in the following order: the possibility of improvement of cardiac function (77.7%), general principles for taking medications (72.7%), actions to take when side effects of medications developed (72.7%), consequences of not following medical advice (72.7%), actions to take in case of worsening symptoms (72.7%) (Table 2). The least frequently reported topics that the patients rated as important or very important were in the following order: the presence of an available support group (38.0%), importance of sharing emotional distress (45.5%), when engaging in sexual activity is allowed (45.5%), strategies to fit in daily weight lifestyle strategies (52.1%), and contributors to the onset of cardiac disease (52.9%).

#### 3.3.2. Topics Addressed in Websites

Heart failure topics that were most frequently addressed in the reviewed websites included symptoms and signs of heart failure (97.0%), prognosis of heart failure (“what happens when someone has heart failure”) (97.0%), causes of heart failure (94.2%), risk factors of heart disease (84.5%), and function and anatomy of the heart (80.0%) (Table 2). However, none of the websites included information related to advice for family members in case of sudden death. A few websites addressed information about advanced directives (1.0%), where family members could learn about cardio-pulmonary resuscitation (1.0%), an available support group (1.0%), normal emotional responses to living with chronic illnesses (1.0%), and the importance of sharing emotional distress (1.9%).

#### 3.3.3. Comparison of Topics Perceived to be Important by Patients and Topics Addressed in the Websites

Of the 46 topics, 39 topics were less likely to be addressed on the websites although patients perceived that the information was important to learn (*p*-values < 0.05) (Table 2). Four topics that were more likely to be addressed on the websites compared to patients’ perceived importance to learn (*p*-values < 0.001) were as follows: symptoms of heart failure, anatomy and function of the heart, contributing factors to heart disease, and prognosis of heart failure.

### 3.4. Heart Literacy Demand

The overall mean PEMAT-understandability score was 58.0% (SD 15.1) ranging from 12.5% to 87.5% (Table 3). Over 90% of the websites included only information related to the main purpose (97.0%), displayed the information in logical order (91.9%), and grouped information into short sections (91.6%). However, only 6% of the websites included a summary of the content, and only 12% defined the medical terms they used. The overall mean PEMAT-actionability score was 19.8% (SD 19.9) ranging from 0.00% to 100%. In addition, 63.3% of the websites suggested at least one action that patients with heart failure could take. However, only 2.0% of the websites provided a concrete tool to help patients with heart failure take action or visual aids for patients to follow the instructions.

### 3.5. Quality

#### 3.5.1. Websites

The appraisal of the quality of the websites is summarized in Table 4 based on the DISCERN criteria. The total DISCERN scores indicated that 33.3% of the websites were rated as very poor quality, 46.5% were poor quality, 18.2% were fair quality, and only 2.0% were good quality. Of the 15 items, the mean scores of nine items were below 2, indicating that they did not fulfill the corresponding quality criterion. The three highest rated items were in the following order: achieved the aims if the websites clearly stated their aims (4.76, SD 0.73), provided additional support and information sources (3.46, SD 1.25), and provided shared decision-making support (3.20, SD 1.90). Items rated as the three lowest were in the following order: the effect of treatment choices on overall quality of life (1.30, SD 0.63), description of risks of each treatment (1.38, SD 0.82), and citations of the sources of information (1.49, SD 0.76).

#### 3.5.2. Comparison by Types of Websites

There were significant differences in website quality by website type (*p*-value = 0.009) (Table 5). Websites created by government or professional organizations had the highest DISCERN total scores, and were significantly different from hospital affiliated and commercial company websites (*p*-values < 0.05).

## 4. Discussion

Searching for health information on the Internet can help patients with heart failure learn about and manage their condition because routine comprehensive patient education is not always readily available in healthcare systems. For example, the average time clinicians spend with patients during a regular office visit is 20 min in the United States and 9 min in Korea [19,20]. A 60-min inpatient education session for heart failure patients is rare [21].

Although online health information can be a good source for patients to supplement what they learn from their clinicians, we found evidence that patients’ expectations about the topics and quality of the information were not met. Our findings show that the reviewed websites mostly included generic information about heart failure, which was not the priority for most patients. In addition, the online heart failure information required a high health literacy level, and the information was rated as poor quality. Websites published by government or professional organizations provided heart failure information requiring relatively low health literacy demand and was rated as higher quality compared to websites by others (e.g., hospitals and commercial companies). The results of this study suggest that online information is not sufficient to fulfill patients’ learning needs, and much of the information is not written so patients can easily understand and take action to manage their condition based on high quality information. Our findings also echo the growing concern about the quality of much of the health information presented online.

We found that online information did not meet the learning needs of patients with heart failure because there was a significant mismatch between what patients wanted to learn and the topics addressed in the websites. Similar results have been found in previous studies showing a disconnect between what topics and information clinicians perceived to be important compared to patients’ perceptions [13,22]. In our study, the topics patients ranked high tended to be related to self-care activities, and especially problems they might encounter such as strategies to use in case their symptoms became worse or they experienced medication side effects. However, these topics were rarely addressed on the websites. This finding implies that patients’ need to develop self-care skills to manage heart failure is not fulfilled online. It also highlights the problem of providing information without incorporating voices of the target population. Therefore, it is crucial for creators of online information to carefully assess what patients with heart failure want to learn.

Both clinicians and patients have ranked psychological topics at the bottom in previous studies [13,22,23,24], even though psychological adjustment is an important part of living with heart failure. In our study, although more than 45% of the patients perceived that managing psychological issues was an important topic to learn, this information was rarely included on the websites (1–3%).

One of the key results of this study is the scarcity of online heart failure information that is written in an understandable and actionable manner. The writing styles, organization, and use of visual aids and numbers were inadequate for patients to read and comprehend the information they needed. However, the most significant problem with the heart failure information from the websites we reviewed was that the information was not actionable, meaning that it was not written in a way that patients could apply what they read to manage their heart failure. This finding is consistent with previous studies showing that actionability of online health information including heart failure is a significant issue [10,25,26]. It is quite concerning that online information may not help patients engage in self-care, as patients with heart failure frequently experience challenges with self-care [27,28].

We also found that the overall quality of the online heart failure information was poor in this study. Cajita and colleagues’ study also found that online heart failure information written in English was only fair quality [10]. Although the scores for most of the items in DISCERN were lower in our study compared to that of Cajita and colleagues, several items were scored low in both studies including items related to citations for the sources of information, the effect of treatment on quality of life, areas of uncertainty, and descriptions about the consequences of no treatment. The overall poor quality of online heart failure information is concerning because patients might not have the skills to evaluate the reliability and quality of the information. Therefore, it is necessary for clinicians to give clear guidelines about how to find credible online information.

Individuals evaluate the credibility of online information based on a variety of factors, such as authority and credibility of the authors, recommendation by others, and references [29]. Although was it was beyond the scope of this study to identify the characteristics of websites with quality online information for readers with a lower health literacy level, we found that websites created by government or professional organizations provided better quality information that was more actionable compared to others. This finding is consistent with the findings of previous studies that have also found that government or professional organizations provided higher quality online information with an adequate level of health literacy demand [10,25]. Although the overall quality and actionability of the information created by government or professional organizations was not satisfactory in our study, guiding patients to search for these types of websites could increase the chance that patients will learn credible and actionable information.

One of the limitations of this study is that the sample was from one country, Korea, and thus only websites written in Korean were included. This sample limits the generalizability of our findings to only online Korean information. In addition, our sample was not representative of the whole heart failure population because the majority of the patient in our sample were male in New York Heart Association functional class I/II.

Another limitation of this study is the exclusion of non-Korean-language websites given our Korean sample. For example, health information on major international cardiology organizations’ websites (e.g., American Heart Association) have a dedicated section for patients and/or their caregivers and have been translated into a very limited number of languages [30]. However, these sites were not included in our study given the nature of our sample: Korean patients. Although we did not evaluate the quality and health literacy demands of the online health information on their websites, it can be assumed that the information is high quality with low health literacy demands since they are reputable and credible sources. Further investigations should evaluate if clinicians should inform patients about these international societies of cardiology as good resources of information.

Some interactive websites also provide information based on patients’ situations (e.g., the duration of heart failure) and a mobile application to support patients’ self-care, but these sites and mobile applications were also not included in this study. Future studies should evaluate these applications given that the popularity of these mobile health applications has increased [3]. Several meta-analyses have reported that they are effective for improving self-care [31,32]. According to the study by Sohn and colleagues [33], more than 60% of patients with heart failure showed interest in using mobile health applications to support self-care. Thus, nurses and other health care providers could develop mobile health applications for patients with heart failure and use them to support patients’ self-care. The data from the applications could also be used when educating patients in follow-up visits. Health care providers should also include content based on patients’ learning needs and evaluate the quality and health literacy demands of the information when they develop these applications.

In addition, although online videos (e.g., YouTube) and mobile applications are another popular online resource, we did not include these resources in this study. However, watching online videos might not be a common practice for older patients with heart failure who are searching for health information because a very small number of older people reported that they knew quite well about such video creation services [34]. We also did not evaluate whether the information on the websites was the most current, evidence-based information. Future research is needed to evaluate whether the online information reflects the most up-to-date evidence.

## 5. Conclusions

Although the Internet is a popular source of health information for patients to learn about their conditions, we found, overall, that the available heart failure information online required high health literacy demand and had relatively poor quality. The sites also tended to include generic heart failure information, so the information did not adequately meet patients’ educational needs about their condition. However, government or professional organizations provided relatively better quality heart failure online information with higher actionability compared to other online information. Clinicians should inform patients with heart failure that the quality of online heart failure information can vary widely and direct them to websites published by government or professional organizations. Clinicians also need to suggest that online publishers (especially, government or professional organizations) include topics that reflect patients’ priorities when they are seeking information about heart failure. Future work is also needed to characterize what constitutes good quality online information with lower health literacy demand so clinicians can provide guidelines on how to search for credible online information.

## Figures and Tables

**Table 1:** Sample characteristics (*n* = 121).

Variables	*n* (%) or Mean (SD)
Age, years (range 25–85)	59 (12.99)
Female	31 (25.6%)
Married or co-habitating	97 (80.2%)
Below high school education	26 (21.5%)
Left ventricular ejection fraction (*n* = 107), %	37.99 (14.10)
Ischemic etiology of heart failure	15 (12.4%)
New York Heart Association functional class I/II	110 (90.9%)
Diuretic	87 (71.9%)
Beta blockers	99 (81.8%)
ACE or ARB	105 (86.8%)

**Table 2:** Comparison of topics perceived as important by patients and topics addressed on the websites.

Items	*n* (%) of the Patients Reported to be Important to Learn (*n* = 121)	*n* (%) of the Websites Addressing the Topics (*n* = 99)	*p*-Value
What symptoms are caused by heart failure?	81 (75.2)	96 (97.0)	<0.001
Can the heart’s function improve?	94 (77.7)	52 (52.5)	<0.001
What to do if I have problems with medications	88 (72.7)	11 (11.1)	<0.001
General rules about taking medications	88 (72.7)	9 (9.1)	<0.001
What can happen if I do not follow my doctor’s recommendations?	88 (72.7)	2 (2.0)	<0.001
What should I do if symptoms worsen?	88 (72.7)	47 (47.5)	<0.001
Why I am short of breath	87 (71.9)	46 (46.5)	<0.001
How significant is my heart failure?	87 (71.9)	37 (37.4)	<0.001
What causes heart failure?	85 (70.2)	93 (93.9)	<0.001
What the heart looks like and how it works	85 (70.2)	77 (77.8)	0.207
What are the signs and symptoms of worsening heart failure?	85 (70.2)	41 (41.4)	<0.001
What effect stress has on my heart	84 (69.4)	11 (11.1)	<0.001
What happens when someone has heart failure?	84 (60.9)	96 (97.0)	<0.001
What my diet restrictions are, if any	83 (68.6)	42 (42.4)	<0.001
How alcohol affects the heart	83 (68.6)	16 (16.2)	<0.001
How to adapt to taking medications every day	82 (67.8)	3 (3.0)	<0.001
How to adapt the recommended diet to my lifestyle	82 (67.8)	14 (14.1)	<0.001
Why I am taking each medication	81 (66.9)	41 (41.4)	<0.001
What the side effects of each medications are	81 (66.9)	17 (17.2)	<0.001
The reason for further testing after I go home	80 (66.1)	13 (13.1)	<0.001
What I can do to improve my heart function	80 (66.1)	65 (65.7)	0.750
What the words sodium, salt and NaCl mean	80 (66.1)	5 (5.1)	<0.001
The signs and symptoms of other heart problems	80 (66.1)	55 (55.6)	0.110
How to adapt the recommended fluid restriction to my lifestyle	79 (65.3)	13 (13.1)	<0.001
What are advanced directives?	79 (65.3)	1 (1.0)	<0.001
When to call the doctor	79 (65.3)	41 (41.4)	<0.001
Where my family can go to learn CPR	78 (64.5)	1 (1.0)	<0.001
What fluid restriction means	78 (64.5)	16 (16.2)	<0.001
What my physical activity restrictions are, if any	78 (64.5)	22 (22.2)	<0.001
What I can do to reduce stress when I go home	77 (63.6)	3 (3.0)	<0.001
The normal emotional response to having a chronic illness	77 (63.6)	1 (1.0)	<0.001
What advice should be given to my family in the event of a sudden death outside the hospital?	77 (63.6)	0 (0.0)	<0.001
How diet affects my heart disease	76 (62.8)	22 (22.2)	<0.001
What my quality of life is expected to be	76 (62.8)	6 (6.1)	<0.001
Why I may not be able to do as much physically as I could before developing heart failure	75 (62.0)	55 (55.6)	0.249
How to tell if I can increase my activity	75 (62.0)	9 (9.1)	<0.001
General rules about eating	73 (60.3)	48 (48.5)	0.079
How these factors affect the heart	72 (59.5)	33 (33.3)	<0.001
General guidelines for physical activity	72 (59.5)	43 (43.4)	0.018
What is my long-term life expectancy?	71 (58.7)	16 (16.2)	<0.001
Why daily weights are needed	68 (56.2)	21 (21.2)	<0.001
Which factors may have contributed to the onset of my heart disease?	64 (52.9)	85 (85.9)	<0.001
How to adapt daily weights to my lifestyle	63 (52.1)	9 (9.1)	<0.001
The importance of talking to someone about my fears, feelings and thoughts	55 (45.5)	2 (2.0)	<0.001
When I can engage in sexual activity	55 (45.5)	4 (4.0)	<0.001
What support groups are available?	46 (38.0)	1 (1.0)	<0.001

**Table 3:** Health literacy demand ratings of online heart failure information (*n* = 99).

Question	Agree (%)
Understandability	
Topic–Content	
1. The material makes the purpose completely evident.	35 (35.4)
2. The material does not include information or content that distracts from the purpose.	96 (97.0)
Topic—Word choice & style	
3. The material uses common, everyday language.	87 (87.9)
4. Medical terms are used only to familiarize the audience with the terms. When used, medical terms are defined.	12 (12.1)
5. The material uses the active voice.	49 (49.5)
Topic—Use of numbers	
6. Numbers appearing in the material are clear and easy to understand. (*n* = 63)	18 (28.6)
7. The material does not expect the user to perform calculations. (*n* = 56)	44 (78.6)
Topic—Organization	
8. The material breaks or “chunks” information into short sections. (*n* = 95)	87 (91.6)
9. The sections have informative headers. (*n* = 92)	73 (73.3)
10. The material presents information in a logical sequence.	91 (91.9)
11. The material provides a summary. (*n* = 95)	6 (6.1)
Topic—Layout & design	
12. The material uses visual cues to draw attention to key points.	54 (54.5)
Topic—Use of visual aids	
15. The material uses visual aids whenever they could to make the content more easily understood	61 (61.6)
16. The visual aids reinforce rather than distract from the content. (*n* = 69)	43 (62.3)
17. The visual aids have clear titles and captions. (*n* = 69)	15 (21.7)
18. The material uses illustrations and photographs that are clear and uncluttered. (*n* = 69)	46 (66.7)
19. The material uses simple tables with short and clear row and column headings. (*n* = 6)	4 (66.7)
Actionability	
20. The material clearly identifies at least one action the user can take.	63 (63.3)
21. The material addresses the user directly when describing actions.	25 (25.3)
22. The material breaks down any action into manageable, explicit steps.	7 (7.1)
23. The material provides a tangible tool whenever it could help the user take action.	2 (2.0)
24. The material provides simple instructions or examples of how to perform calculations. (*n* = 9)	3 (33.3)
25. The material explains how to use the charts, graphs, tables, or diagrams to take action. (*n* = 1)	1 (100.0)
26. The material uses visual aids whenever they could make it easier to act on the instructions.	2 (2.0)

**Table 4:** Quality rating of online heart failure information (*n* = 99).

Question	Score (SD)
Reliability	
1. Are the aims clear?	1.62 (0.71)
2. Does it achieve its aims? (*n* = 53)	4.76 (0.73)
3. Is it relevant?	1.97 (1.16)
4. Is it clear what sources of information were used to compile the publication other than author or producer?	1.49 (0.76)
5. Is it clear when the information used or reported in the publication was produced?	2.24 (0.97)
6. Is it balanced and unbiased?	2.41 (1.20)
7. Does it provide details of additional sources of support and information?	3.46 (1.25)
8. Does it refer to areas of uncertainty?	1.65 (0.84)
Quality of information	
9. Does it describe how each treatment works?	1.87 (0.88)
10. Does it describe the benefits of each treatment?	2.33 (1.40)
11. Does it describe the risks of each treatment?	1.38 (0.82)
12. Does it describe what would happen if no treatment is used?	1.79 (1.17)
13. Does it describe how the treatment choices affect overall quality of life?	1.30 (0.63)
14. Is it clear that there may be more than one possible treatment choice?	1.80 (0.94)
15. Does it provide support for shared decision-making?	3.20 (1.90)
**Total scores**	31.07 (8.96)
Overall quality rating	
16. Based on the answers to all of the above questions, rate the overall quality of the publication as a source of information about treatment choices	2.51 (0.68)

**Table 5:** Comparison of health literacy demand and quality of online heart failure information by website type.

Types of Websites	Patient Education Material Evaluation Tool for Print Materials		Discern
	Understandability	Actionability	
Hospital or clinic (*n* = 70)	58.94 (15.33)	17.95 (17.86) ^b^	30.71 (8.38) ^b^
Commercial company (*n* = 15)	54.32 (12.24)	16.44 (15.51) ^b^	27.93 (9.21) ^b^
Physician (*n* = 7)	53.21 (19.72)	14.29 (15.12) ^b^	31.00 (8.19)
Government or professional organization (*n* = 7)	60.87 (13.53)	50.95 (28.20) ^a^	41.43 (9.41) ^a^
Overall *p*-values	0.009	0.553	<0.001

Note. Different superscripts indicate significant differences among the groups. Values are the mean (SD).

## Data Availability

The data presented in this study are available on request from the corresponding author.

## Abbreviation

HFPLNI: Heart Failure Patients’ Learning Needs Inventory.
